# Hypomagnetic Field Induces the Production of Reactive Oxygen Species and Cognitive Deficits in Mice Hippocampus

**DOI:** 10.3390/ijms23073622

**Published:** 2022-03-26

**Authors:** Lanxiang Tian, Yukai Luo, Aisheng Zhan, Jie Ren, Huafeng Qin, Yongxin Pan

**Affiliations:** 1 Biogeomagnetism Group, Key Laboratory of Earth and Planetary Physics, Institute of Geology and Geophysics, Chinese Academy of Sciences, Beijing 100029, China; luoyukai19@mails.ucas.ac.cn (Y.L.); zhanaisheng18@mails.ucas.ac.cn (A.Z.); renjie212@mails.ucas.ac.cn (J.R.); yxpan@mail.iggcas.ac.cn (Y.P.); 2 Innovation Academy for Earth Science, Chinese Academy of Sciences, Beijing 100029, China; huafeng_qin@mail.iggcas.ac.cn; 3 The Paleomagnetism and Geochronology Laboratory, Institute of Geology and Geophysics, Chinese Academy of Sciences, Beijing 100029, China; 4 University of Chinese Academy of Sciences, Beijing 100049, China

**Keywords:** hypomagnetic field, cognitive dysfunction, hippocampus, reactive oxygen species, redox balance gene expression, oxidative stress

## Abstract

Previous studies have found that hypomagnetic field (HMF) exposure impairs cognition behaviors in animals; however, the underlying neural mechanisms of cognitive dysfunction are unclear. The hippocampus plays important roles in magnetoreception, memory, and spatial navigation in mammals. Therefore, the hippocampus may be the key region in the brain to reveal its neural mechanisms. We recently reported that long-term HMF exposure impairs adult hippocampal neurogenesis and cognition through reducing endogenous reactive oxygen species (ROS) levels in adult neural stem cells that are confined in the subgranular zone (SGZ) of the hippocampus. In addition to adult neural stem cells, the redox state of other cells in the hippocampus is also an important factor affecting the functions of the hippocampus. However, it is unclear whether and how long-term HMF exposure affects ROS levels in the entire hippocampus (i.e., the dentate gyrus (DG) and ammonia horn (CA) regions). Here, we demonstrate that male C57BL/6J mice exposed to 8-week HMF exhibit cognitive impairments. We then found that the ROS levels of the hippocampus were significantly higher in these HMF-exposed mice than in the geomagnetic field (GMF) group. PCR array analysis revealed that the elevated ROS levels were due to HMF-regulating genes that maintain the redox balance in vivo, such as *Nox4*, *Gpx3*. Since high levels of ROS may cause hippocampal oxidative stress, we suggest that this is another reason why HMF exposure induces cognitive impairment, besides the hippocampal neurogenesis impairments. Our study further demonstrates that GMF plays an important role in maintaining hippocampal function by regulating the appropriate endogenous ROS levels.

## 1. Introduction

Geomagnetic fields, which already existed before life appeared on Earth [1,2], are one of the important environmental conditions for the origin, evolution, survival, and development of living organisms on Earth [3,4]. Many kinds of animals use the information of GMF for orientation and long-distance navigation [5,6,7]. The major effects of geomagnetic fields on organisms include shielding from radiation from solar winds and other cosmic high-energy particles, preventing oxygen escape, and maintaining the habitable environment for organisms [8]. The decrease in geomagnetic field intensity in the late Quaternary affected the evolution of humans and large mammals [9,10]. Furthermore, the elimination of GMF, the so-called hypomagnetic field (HMF, with intensity < 5 μT), is also a risk factor to the health of astronauts in outer space [11]. Changes in GMF intensity have been shown to affect the biological processes of modern organisms [12]. Previous studies suggested that the absence of GMF would reduce learning and memory ability in one-day-old chicks and fruit flies, even humans [13,14,15]. However, the underlying neural basis of cognitive dysfunctions in animals remains poorly understood.

Animal learning and cognitive behavior are highly dependent on the hippocampal region of the brain [16,17,18], and the hippocampus is also a key magnetoreception brain region [19,20,21]. Therefore, the potential molecular mechanisms of cognitive dysfunctions in HMF-exposed animals can be explored from the hippocampus. The hippocampus mainly contains the DG and CA regions, and the DG has three distinct layers, including an outer molecular layer, a middle granule cell layer, and an inner polymorphic layer (hilus). In addition, there is a SGZ between the granule cell layer and the polymorphic layer. The SGZ maintains neural stem cells and neurogenesis into adulthood; these play a major role in cognitive function [22,23,24]. Our recent study showed that long-term HMF exposure significantly impairs adult hippocampal neurogenesis and cognition through decreasing endogenous ROS levels in adult neural stem cells in the SGZ of mice [25]. Intracellular oxidative stress is also known to play an important role in cognitive dysfunction besides the impairment of hippocampal neurogenesis [26,27,28]. However, the effect of the HMF on ROS levels throughout the hippocampus in vivo remains unknown.

ROS are by-products of aerobic metabolism, which include the superoxide anion (O_2_^•−^), hydrogen peroxide (H_2_O_2_), and hydroxyl radicals (^•^OH). A small increase in ROS levels is required for the activation of signaling pathways to initiate biological processes, while high levels of ROS can cause oxidative stress that results in damage to DNA, protein, or lipids [29,30,31]. Oxidative stress is generated by an imbalance between the production of ROS and the antioxidant defense against ROS in cells and tissues; this has been linked to cancer, cardiovascular and metabolic diseases, and several neurological diseases (i.e., Parkinson’s disease, Alzheimer’s disease), depression, and memory loss [32,33,34,35]. Previous studies showed that environmental stressors (i.e., ionizing radiation, ultraviolet light, and heavy metals) cause a large increase in ROS production, therefore leading to cell and tissue damage (oxidative stress) [36,37,38]. The shielding or absence of GMF also affects the ROS levels in cells in in vitro experiments. Specifically, the ROS levels of mouse primary skeletal muscle cells were largely increased after shielding GMF for three days [39],while HMF exposure can also decrease ROS production, as has been described in human neuroblastoma SH-SY5Y cells and other cell lines [40,41]. The reasons for these different results may be related to magnetic field strength, cell types, and exposure time.

In this study, we sought to determine whether and how long-term HMF exposure affect endogenous ROS levels throughout the hippocampus in mice. We found that 8-week HMF exposure induced anxiety in mice, impaired cognitive function, and increased ROS levels in both the hippocampal DG and CA regions. Compared to the GMF group mice, four key genes related to oxidative stress were upregulated, while two genes related to antioxidants were downregulated in the hippocampus of HMF-exposed mice, as revealed by real-time PCR arrays analysis. These results indicated that HMF modulated the key genes expression of the oxidative stress pathway to induce oxidative stress in hippocampal cells. Since high levels of ROS may cause oxidative stress in the hippocampus, we suggest that this is another reason for the HMF inducing cognitive dysfunction, besides the impairment of hippocampal neurogenesis. Our study further demonstrates that GMF plays an important role in maintaining hippocampal function by regulating the appropriate endogenous ROS levels. It provides a better understanding of the underlying molecular mechanisms of cognitive deficits in mice in an HMF environment.

## 2. Results

### 2.1. The Hypomagnetic Field Exposure Impaired Cognitive Function in Mice 

**Figure 1:**
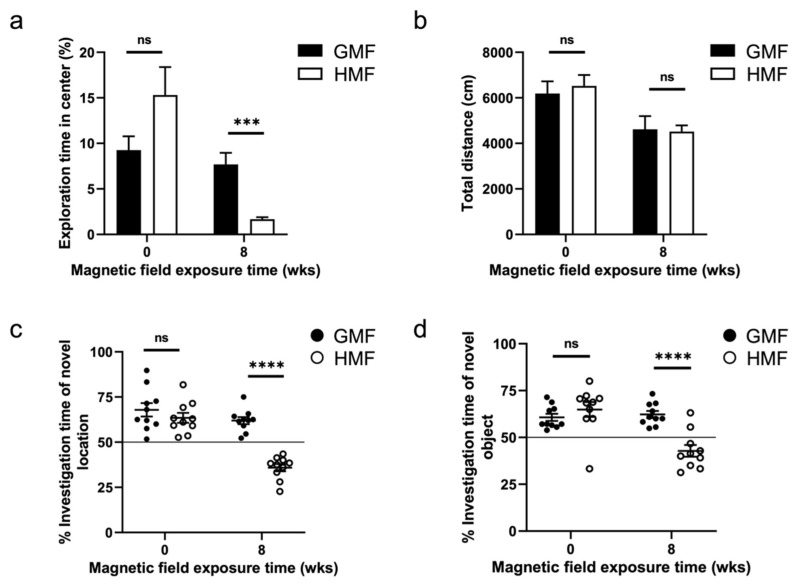
Results of Open-Field, OLT, and NOR tests in GMF- and HMF- exposed mice (*n* = 10/group). (**a**) The percent time spent in the center of the open field in mice at 0 w and 8 w GMF/HMF exposure. *** *p* = 0.0002, unpaired *t*-test. (**b**). The total traveled distances in the open field test in mice at 0 w and 8 w GMF/HMF exposure. (**c**) The percentage of time spent exploring the novel location in mice at 0 w and 8 w GMF/HMF exposure. **** *p* < 0.0001, unpaired *t*-test. (**d**) The percentage of time spent exploring a novel object in mice at 0 w and 8 w GMF/HMF exposure. **** *p* < 0.0001, unpaired *t*-test. Data are presented as mean ± SEM. n.s. = not significant.

We performed behavioral tests, including open field, object location test (OLT) and novel object recognition (NOR), before (0 w) and after (8 w) magnetic field exposure. The body weight between the HMF-exposed mice and GMF-exposed mice was not different (*p* > 0.05) at 0 w and 8 w magnetic field exposure (Figure S1). There were no behavioral differences between the GMF and HMF groups, randomly divided before the magnetic field exposure (0 w), but after 8 weeks of magnetic field treatments, the time spent in the center region of the open field, the exploration time in new object/new position in HMF mice was significantly reduced compared to the GMF mice (Figure 1). In the open field test, GMF mice acclimated to the chamber and eventually explored the center area, while HMF mice spent significantly less time in the open area and more time closer to the walls (*p* = 0.0002). The results indicated that HMF-exposed mice exhibited anxiety after 8-week HMF exposure (Figure 1a). However, the total traveled distances of HMF-exposed mice and GMF-exposed mice were not different (*p* > 0.05). The results suggested that the general activity of mice was not affected by HMF exposure (Figure 1b). In the OLT and NOR tests, GMF mice spent relatively more time exploring the new object/new location, while the time exploring the new object/new location of HMF mice was significantly reduced (*p* < 0.0001) (Figure 1c,d). These data suggested that the HMF exposure impaired the spatial and cognitive memory of mice.

### 2.2. The Hypomagnetic Field Increased ROS Levels in DG and CA Regions in Hippocampus 

**Figure 2:**
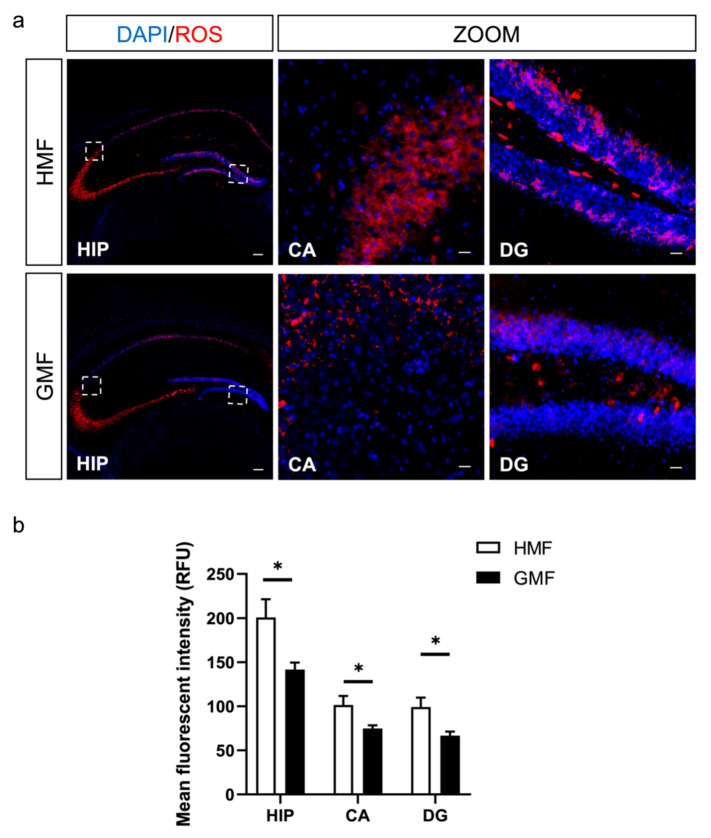
The effects of HMF treatment on ROS levels in hippocampus. (**a**) Representative images of hydroethidine fluorescence (red) in the DG and CA of the hippocampus (HIP) in GMF- and HMF-exposed mice. DAPI staining (blue) was used for distinguishing the different hippocampal regions in brain sections. HIP Scale bar = 500 μm, CA/DG Scale bar = 100 μm. (**b**) Quantitative analyses of hydroethidine fluorescence intensity (ROS) levels of neural cells in the dentate gyrus, cornu ammonis areas, and both hippocampus of GMF- and HMF-exposed mice. *n* = 4 mice. Data are presented as mean ± SEM. * *p* < 0.05. HIP: *p* = 0.0374, unpaired *t*-test. CA: *p* = 0.0481, unpaired *t*-test. DG: *p* = 0.0301, unpaired *t*-test.

To directly measure the cellular ROS content in the hippocampus in vivo, we performed in situ ROS labeling by injecting ROS-sensitive dye hydroethidine into adult mice. As shown in Figure 2, the hippocampus presents intact DG and CA shapes under GMF and HMF conditions by DAPI staining (blue). The endogenous ROS levels were measured using hydroethidine fluorescence (red). Compared to GMF-exposed mice, the endogenous ROS levels were significantly increased in the DG and CA areas of the hippocampus in the HMF-exposed mice (Figure 2b). The significant elevation of ROS levels mainly occurred in the outer molecular layer, partly in the granule cell layer (Figure 2a). 

### 2.3. Differentially Expressed Genes Associated with Oxidative Stress

Compared to the GMF group, four key genes related to oxidative stress were upregulated, while two genes related to antioxidants were downregulated in the hippocampus of HMF-exposed mice. The upregulated genes were NADPH oxidase 4 (*Nox4*), eosinophil peroxidase (*Epx*), Keratin 1 (*Krt1*), and Nitric oxide synthase 2 (*Nos2*). Functionally, these upregulated genes are basically related to ROS and NO generation and metabolism. The downregulated genes were glutathione peroxidase 3 (*Gpx3*) and heat shock protein 1A (*Hspa1a*), which are associated with the antioxidants and cytoprotective functions (see Table 1). Especially compared to the GMF group, the fold change value of *Nox4* expression is 2.55. *Nox4* encodes a member of the *Nox* enzyme family, which is a major source of oxidative stress in cells. Our results show that the elimination of the geomagnetic field causes *Nox4* expression to increase, while the antioxidant glutathione peroxidase3 decreases; the imbalance leads to increased ROS production in the hippocampal tissue cells.

## 3. Discussion 

The behavioral data in this study demonstrates that long-term HMF exposure induces anxiety and cognitive impairments in mice. These behavioral results are similar to those of previous studies. For example, short-term HMF-exposure (72 h) also induced a significant increase in anxiety-related behaviors, as shown in a previous study [42]. The effect of HMF exposure on cognitive impairments in mice is also identical to our previous report [25]. Further, in situ ROS labeling results clearly showed that long-term HMF exposure increased cellular ROS in the DG and CA regions of the hippocampus of mice (Figure 1 and Figure 2), different from the low ROS levels found in the hippocampal adult neural stem cells [25]. High ROS levels have been known to be associated with oxidative stress, aging, and brain dysfunction [43,44,45]. Anxiety behavior has also been linked to oxidative stress in animals [46,47]. Therefore, we suggested that HMF might induce oxidative stress in the hippocampus by increasing the ROS levels, thus causing anxiety and cognitive dysfunction behaviors in mice.

**Figure 3:**
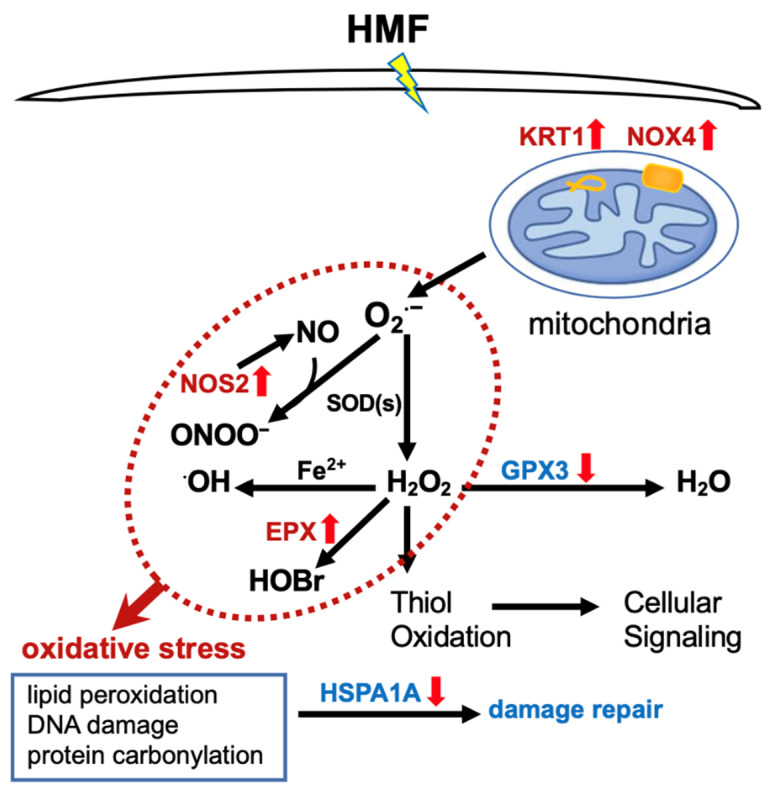
Schematic diagram of how the hypomagnetic field increased ROS production to induce oxidative stress by regulating several key gene expressions.

How does HMF exposure affect ROS levels in mice hippocampus? Our data suggest that the HMF regulates several key gene expressions associated with the oxidative stress pathway (Table 1). The intracellular ROS levels are dependent on the dynamic balance between ROS generation and elimination. From our results, we drew a schematic diagram to illustrate how the hypomagnetic field regulates ROS levels (see Figure 3). On one hand, mitochondria and NADPH oxidases are two main sources of ROS production in cells. We found that the mRNA expression of *Nox4* was upregulated after HMF exposure in vivo, which would increase the mitochondrial ROS production in cells [48,49,50]. Some studies have also indicated that the upregulation of *Nox4* is prone to mitochondrial dysfunction [48,51]. Meanwhile, the *Epx* mRNA expression in hippocampal cells also increased after HMF exposure, which uniquely metabolizes H_2_O_2_ into highly reactive and destructive hypobromous acid [52]. In addition, *Nos2* mRNA expression also increased, which encodes a nitric oxide synthase to generate nitric oxide. Nitric oxide, a gaseous signaling molecule, plays important roles in the physiology and pathophysiology of the central nervous system, depending on its concentration. At low concentrations, NO is neuroprotective, but beyond certain concentrations, it is cytotoxic and associated with neurodegenerative disorders [53,54,55]. It often reacts with O_2_^•−^ to yield strong reactive nitrogen species (RNS) such as peroxynitrite (ONOO^−^) [56]. Therefore, compared to the GMF group mice, the upregulation of the genes associated with ROS and RNS generation finally leaded to an increase in ROS and RNS production. On the other hand, the redox control in cells is the superoxide-metabolizing enzymes, superoxide dismutases (SODs), and the H_2_O_2_/peroxide-metabolizing enzymes, catalase, glutathione peroxidases (GPXs), peroxiredoxins (PRDXs), and glutaredoxins (GRXs). Superoxide dismutases, which rapidly convert O_2_^∙−^ to H_2_O_2_, had no significant difference in expression levels between the HMF and GMF groups in this study, while *Gpx3* mRNA expression was greatly decreased after HMF exposure in vivo (Table 1). Glutathione peroxidase tightly regulates and controls the intracellular H_2_O_2_ levels [29,31,32,33]. In normal physiological conditions, the antioxidants reduce hydrogen peroxide to water to limit its harmful effects. Under HMF exposure, the low expression of the antioxidant defense system greatly affects their detoxification function, resulting in excessive hydrogen peroxide (Table 1 and Figure 3). The latter reacts readily with ferrous iron to produce a reactive hydroxyl radical [57]. Furthermore, the mRNA expression of heat shock protein (HSP) also decreased in this study. The HSP response is generally concerned with heat shock, which displays remarkable roles inside cells under a variety of stresses, including oxidative stress and radiation and recognizing and assisting in unfolded or misfolded protein restructuring [58]. The epithelial intermediate filament protein keratins (KRT 1) regulate both the structural and dynamic functions of cellular mitochondria, which comprise the intermediate filaments of the cytoskeleton [59,60]. Mitochondrial reactive oxygen species are required for the hypoxia-induced degradation of keratin intermediate filaments [61]. Whether the upregulation of keratin gene expression involves changes in mitochondrial shape and function in this study is still unknown. These results suggested that long-term HMF exposure broke the balance of reactive oxygen species production and elimination, leading to high ROS levels in the hippocampus.

In this study, we found, for the first time, that the influences of the HMF on ROS levels in adult neural stem cells and other hippocampal cells were different in vivo. The reasons may be related to the cell types [40,41] and the rate of ROS production varying across brain regions [62]. Previous studies have shown that ROS have a dual role, redox signaling and oxidative stress [63,64]. Appropriate levels of ROS act as a signaling role to influence signal transduction by thiol oxidation [30,65,66]. However, high levels of ROS are closely associated with oxidative stress, aging, and brain dysfunction [43,44,45]. Similar results regarding increased ROS production and oxidative stress were also shown in many animal studies of the biological effects of electromagnetic fields (EMFs) from power systems [67,68]. These studies indicate that HMF or EMF may contribute to the production of ROS and cause oxidative stress, which could further trigger or enhance neurodevelopmental abnormalities or cancer. For example, previous studies have shown that oxidative stress may cause mitochondrial impairment and lipid peroxidation [37], and mitochondrial dysfunction is one of the major features of biological responses to spaceflight, which is also heavily linked to oxidative stress [69]. Moreover, the upregulation of *Nox4*, as shown in our study, is also prone to mitochondrial dysfunction [48,51]. Therefore, further quantitative investigations of the damage or dysfunction of organelles and biological macromolecules caused by oxidative stress are necessary to better assess the human health risk. In the next step, we will aim to investigate the effects of HMF exposure on the metabolic function of mitochondria and the activity of peroxide oxidation of lipids in the hippocampus of mice in vivo. Furthermore, in order to test whether the effect of HMF exposure on the hippocampus is universal or species-specific, we will use female mice and other strains of mice or even other species in future studies.

## 4. Materials and Methods

### 4.1. Animals and Magnetic Fields Exposure

Adult 7-week-old male mice (C57BL/6J) were purchased and housed in the experimental coils to acclimate to the exposure environments for one week; then, we started the hypomagnetic field/geomagnetic field exposure experiments. The mice were randomly allocated to GMF and HMF exposure groups. All animals were housed with a constant temperature (23 ± 1 °C) with a 12:12 h light: dark cycle. Water and food were given ad libitum. The number of animals used for the behavioral test was 10 individuals in each group. Four animals in each group were used for immunofluorescence analysis, and three animals in each group were used for PCR arrays analysis. The experimental hypomagnetic field environment was simulated using a double-wrapped coil system (Figure S1a), which was set up in the laboratory of the Beijing National Observatory of Space Environment, at the IGG, for the uniform, stable geomagnetic field background. All animal experiments were performed double-blindly. All procedures and husbandry were performed according to protocols approved by the Institutional Animal Care and Use Committee at the Institute of Geology and Geophysics (IGG), Chinese Academy of Science. The magnetic strength and electromagnetic field inside the cages of the HMF and GMF environments were measured by Mag-13MS sensors combined with a Spectramag-6 instrument (Bartington Instruments Limited, Witney, UK) during the experiments. The magnetic field intensity of the HMF was about 31.9 ± 4.5 nT during the experimental duration. For GMF control, the magnetic field intensity was 55,548.5 ± 12.8 nT (mean ± SEM) (Figure S1b). The square root of power spectral density (PSD) of the ambient magnetic field at frequencies ranging from 5 to 100 Hz inside the cages of GMF and HMF environments is shown in Figure S1c. The 50 Hz power frequency peak inside the GMF and HMF cages was about 0.06 and 0.83 nT/Hz^1/2^, respectively. The temperature, humidity, noise levels, and light intensity inside the cages of the GMF and HMF environments were also monitored (Figure S1d–g). The body weight was comparable between GMF- and HMF-exposed mice during the experimental duration (Figure S2). 

### 4.2. Behavioral Tests

Behavioral analyses were carried out in GMF- and HMF- exposed mice (*n* = 10) at 0 and 8 weeks after magnetic field exposure.

#### 4.2.1. Open-Field Test

In order to assess the anxiety-like and activity levels of the animals, the open-field test was performed as previously described [70,71]. After preparing the open field apparatus and software, mice were allowed to acclimate to the procedure room for a minimum of 30 min before starting the test. A single mouse was placed in the middle of the open field maze while concurrently activating the SMART software to begin tracking mouse movement for 10 min. At the end of the test period, the mouse was removed from the open field apparatus and returned to its home cage. To remove the olfactory cues from the previous mouse, the apparatus was cleaned with 70% ethanol. The locomotor activity of the mice was analyzed with Panlab SMART 3.0 Software. 

#### 4.2.2. OLT or NOR Test

In order to evaluate the spatial and cognitive memory of mice, the OLT and NOR were performed in the open-field-testing arena. Both of these tests exploit the inherent preference of mice for novelty to reveal the memory for previously encountered objects; the OLT primarily evaluates spatial learning, which relies heavily on hippocampal activity. The NOR, in contrast, evaluates the non-spatial learning of object identity, which relies on multiple brain regions. The experimental protocol was followed as previously described [72]. Novel object or location preference is expressed as the percentage of time spent exploring the novel object or location compared with the cumulative time spent exploring both objects. A value above 50% indicates a greater investigation of the novel location or object. The same groups of mice underwent every test. The OLT test was performed first; the NOR experiment was carried out later. 

### 4.3. Immunofluorescence Analysis of the Endogenous ROS Levels

In order to measure the endogenous ROS levels, mice were intraperitoneally injected with the ROS-sensitive dye dihydroethidium (HEt, 25 mg/kg; Sigma-Aldrich Co., Saint Louis, MO, USA, 37291) at 4 h prior to perfusion-fixation. Then, mice were deeply anesthetized via isoflurane inhalation and then transcardially perfused with saline followed by 4% paraformaldehyde (PFA) after 8-week HMF and GMF exposures. Brains were dissected and post-fixed in 4% PFA overnight and then equilibrated in 30% sucrose buffer. Brains were sectioned coronally with a freezing microtome (Leica SM2400) into 40 µm thick sections. Serial sections were stored in 96-well plates filled with cryoprotectant solution (glycerol, ethylene glycol, and 0.1 M phosphate buffer, pH 7.4, 1:1:2 by volume) in a −20 °C freezer. To minimize biased sample collection, 1 in 12 serial sections, starting at the beginning of the hippocampus (relative to bregma, −1.22 mm) to the end of the hippocampus (relative to bregma, −3.88 mm), were used. For measurement of relative CA/DG volume, the tissue sections were stained with DAPI (Sigma-Aldrich, #D9542), and the staining sections were mounted, coverslipped, and then maintained at 4 °C in the dark until imaging. Images were acquired on an Olympus FV1000 multiphoton confocal system with a multitrack configuration. The images (40×) were spliced into an overall hippocampal field of view, and the fluorescent intensity of sections was analyzed by using ImageJ software (NIH, Bethesda, MD, USA). The average fluorescence intensity was calculated by dividing the total fluorescence intensity of the specific circled hippocampal region by the area.

### 4.4. PCR Arrays Analysis 

To decipher the molecular mechanism underlying the influence of HMF exposure on the endogenous ROS levels in the hippocampus, we analyzed gene expression for key genes involved in the mouse oxidative stress by real-time PCR arrays analysis. The RT2 Profiler PCR Array of “mouse oxidative stress” (PAMM-065ZA, Qiagen, Valencia, CA, USA) was used in the study. It profiles the expression of 84 genes related to oxidative stress; for example, peroxidases are represented on this array, including glutathione peroxidases (GPx) and peroxiredoxins (TPx). Also included are the genes involved in ROS metabolism, such as oxidative stress-responsive genes, and genes involved in superoxide metabolism, such as SODs. The experiments followed the manufacturer’s instructions. Hippocampus samples of mice were carefully isolated under an anatomical microscope and immediately frozen at −80 °C. Total RNA was extracted using an RNA extraction kit according to the manufacturer’s instructions. RNA quality was determined using the NanoDrop 2000 (Thermo Scientific Inc., Wilmington, DE, USA). About 1 μg total RNA was reverse transcribed using the RT2 First-Strand Kit (Cat. No. 330401; Qiagen). The cDNA was used on the real-time RT2 Profiler PCR Array (Cat. no. PAMM-065Z; Qiagen) in combination with RT2 SYBR Green ROX qPCR Mastermix (Cat. No. 330623; Qiagen). The data analysis was performed on the QIAGEN web portal at GeneGlobe (http://www.qiagen.com/geneglobe (accessed on 19 January 2021)). The relative expression of each mRNA was normalized using the equation 2^−^^∆∆Ct^. Significantly altered expression was determined only if it displayed a fold-regulation higher/less than 1.5, with a *p*-value < 0.05. Gene expression was related to the mean expression of housekeeping genes included in the array.

### 4.5. Statistical Analysis

An unpaired *t*-test was employed to determine the differences between the GMF and HMF groups. Data were valued within a confidence interval of 95%. A *p*-value of less than 0.05 was considered statistically significantly different (*p* < 0.05).

## Figures and Tables

**Table 1:** Differentially expressed genes in HMF group mice vs. GMF group mice.

Gene Symbol	Gene Full Name	Fold Regulation
*Nox4*	NADPH oxidase 4	2.55
*Epx*	eosinophil peroxidase	1.85
*Krt1*	keratin 1	1.86
*Nos2*	nitric oxide synthase 2	1.60
*Gpx3*	glutathione peroxidase 3	−1.70
*Hspa1a*	heat shock protein 1A	−1.64

## Data Availability

Our data presented in this study are available upon request from the corresponding author.

## Supplementary Materials

The following supporting information can be downloaded at: https://www.mdpi.com/article/10.3390/ijms23073622/s1.
