# Supplementary material for: Hypomagnetic Field Induces the Production of Reactive Oxygen Species and Cognitive Deficits in Mice Hippocampus
Source: Int J Mol Sci. 2022 Mar 26;23(7):3622. doi: 10.3390/ijms23073622 (PMC8998670; doi:10.3390/ijms23073622)
Supplement: Supplementary file 1 [file ijms-23-03622-s001.zip › ijms-1634210-supplementary.pdf]

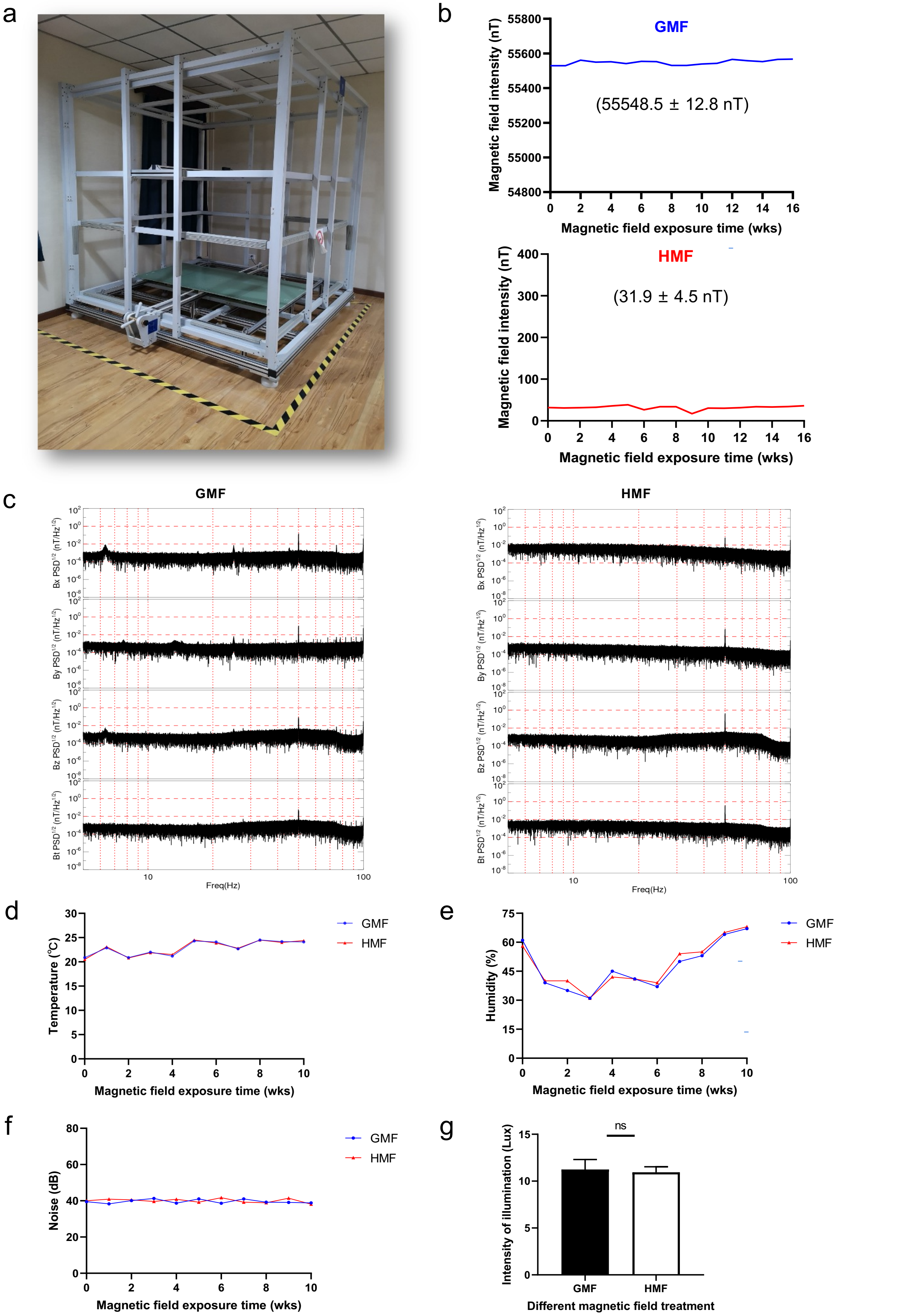

Figure S1. Experimental apparatus and environmental parameters in GMF and HMF. (a) A photo of the experimental apparatus for HMF, same as the GMF apparatus. (b) Magnetic field intensities of GMF and HMF in experimental duration. (c) The square root of power spectral density (PSD) of the ambient magnetic field at frequencies ranging from 5Hz to 100 Hz inside the cages of GMF and HMF environments. (d-g) The temperature, humidity, noise levels, and light intensities inside the cages of GMF and HMF environments. Data presented as mean ± SEM, P=0.8098, unpaired t-test. n.s. = not significant.

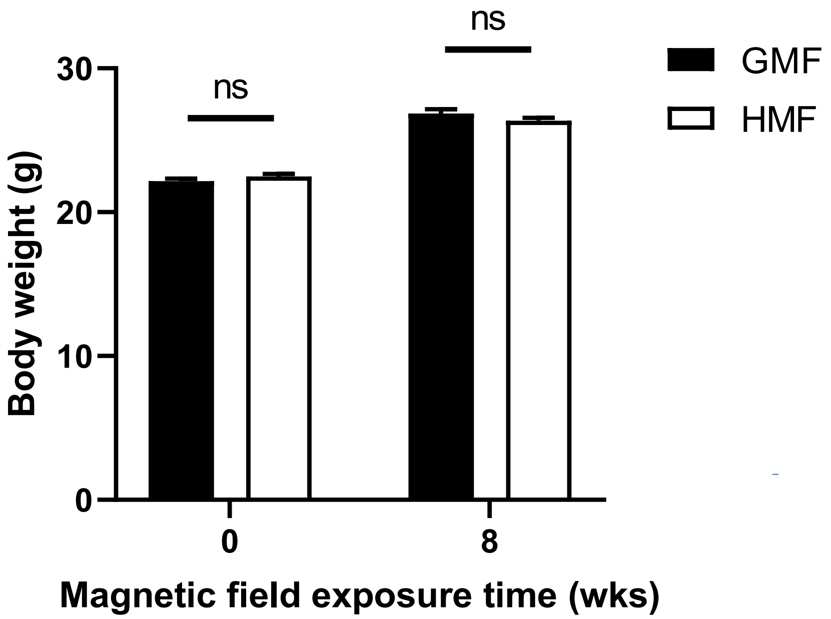

Figure S2. The body weight of mice during GMF- or HMF-exposure (GMF, n = 10 mice, HMF, n = 10 mice, *P* (0w) =0.2083, *P* (8w) =0.2259, unpaired t-test. n.s. = not significant).
